# Natural environmental water sources in endemic regions of northeastern Brazil are potential reservoirs of viable *Mycobacterium leprae*

**DOI:** 10.1590/0074-02760170117

**Published:** 2017-12

**Authors:** Maria Luisa Bezerra de Macedo Arraes, Maísa Viana de Holanda, Luana Nepomuceno Gondim Costa Lima, José Antônio Beltrão Sabadia, Cynthia Romariz Duarte, Rosa Livia Freitas Almeida, Carl Kendall, Ligia Regina Sansigolo Kerr, Cristiane Cunha Frota

**Affiliations:** 1 Universidade Federal do Ceará, Faculdade de Medicina, Departamento de Patologia e Medicina Legal, Fortaleza, CE, Brasil; 2 Instituto Evandro Chagas, Seção de Bacteriologia e Micologia, Belém, PA, Brasil; 3 Universidade Federal do Ceará, Departamento de Geologia, Fortaleza, CE, Brasil; 4 Universidade de Fortaleza, Programa de Pós-Graduação em Saúde Coletiva, Fortaleza, CE, Brasil; 5 Tulane University, School of Public Health and Tropical Medicine, Department of Global Community Health and Behavioral Sciences, New Orleans, LA, USA; 6 Universidade Federal do Ceará, Faculdade de Medicina, Departamento de Saúde Comunitária, Fortaleza, CE, Brasil

**Keywords:** Mycobacterium leprae, water, mRNA, leprosy, disease transmission

## Abstract

**BACKGROUND:**

The detection of live *Mycobacterium leprae* in soil and animals other than humans suggests that the environment plays a role in the transmission of leprosy.

**OBJECTIVE:**

The objective of this study was to investigate the presence of viable *M. leprae* in natural water sources used by the local population in five municipalities in the state of Ceará, northeastern Brazil.

**METHODS:**

Samples were collected from 30 different sources. Viable bacilli were identified by reverse transcriptase polymerase chain reaction (PCR) of the *M. leprae gyrA* gene and sequencing of the PCR products. Physicochemical properties of each water source were also assessed.

**FINDINGS:**

*M. leprae gyrA* mRNA was found in 23 (76.7%) of the water sources. No association was found between depth of the water and sample positivity, nor was there any association between the type of water used by the population and sample positivity. An association between viable *M. leprae* and temperature and pH was found. Georeferencing showed a relation between the residences of leprosy cases and water source containing the bacterium.

**MAIN CONCLUSIONS:**

The finding of viable *M. leprae* in natural water sources associated with human contact suggests that the environment plays an important role in maintaining endemic leprosy in the study region.

Leprosy is a public health problem, especially in developing countries. The main mode of transmission of *Mycobacterium leprae,* the leprosy-causing agent, is inhalation of infectious aerosols released by untreated cases with the multibacillary (MB) clinical form of the disease. However, the detection of live bacilli in the peridomiciliary soil of cases suggests that the environment may also play a role in the disease transmission (Turankar et al. 2016). Other studies have identified possible non-human sources of the bacillus (Truman & Fine 2010), including water (Wahyuni et al. 2010), plants (Mostafa et al. 1995), armadillos (Frota et al. 2012, Kerr et al. 2015), primates (Gormus et al. 1998), and insects (Neumann et al. 2016).

The role of the environment as a route of disease transmission is supported by reports of leprosy cases with no history of previous contact with another case (Marcos et al. 2015), the detection of new cases close to sources of water (Kerr-Pontes et al. 2006), and high seropositivity to anti-phenolic glycolipid-l (PGL 1) in communities with no leprosy cases (Frota et al. 2010).

Brazil, India, and Indonesia account for 81% of all new cases identified globally each year (WHO 2015). In Ceará state, northeastern Brazil, leprosy remains endemic, with the rate of detection of new cases in children under 15 years of age increasing slightly, from 5.4/100,000 inhabitants in 2010 to 6.1/100,000 inhabitants in 2014. In Ceará, in 2015, 80.5% of the municipalities registered new cases of the disease (SESA 2016).

The use of molecular techniques to detect nucleic acids and methodologies that inactivate amplification inhibitors has permitted the detection of mRNA in environmental samples. Because *M. leprae* does not grow in axenic media, detection of mRNA from specimens has been employed to determine the presence of viable *M. leprae* (Davis et al. 2013). In mycobacteria, ribosome degradation is one of the first ultrastructural signs of loss of viability; it precedes bacteriolysis (Silva et al. 1987). However, the half-life of mRNA varies for each transcript, and it is also related to RNase E activity and bacterial adaptation to changes in growth conditions (Esquerre et al. 2015). Therefore, the survival time of *M. leprae* mRNA remains unknown.

In the present study, we investigated the presence of viable *M. leprae* in environmental water samples from five municipalities in the state of Ceará: Boa Viagem, Crato, Juazeiro do Norte, Mulungu, and Sobral. We extracted mRNA from the environmental samples and then perform reverse transcriptase-polymerase chain reaction (RT-PCR) amplification and sequencing of *gyrA.*

## MATERIALS AND METHODS

*Description of the study areas* - In this study, water samples from the municipalities of Boa Viagem, Crato, Juazeiro do Norte, Mulungu, and Sobral, located in the state of Ceará in the northeastern region of Brazil, were assessed. All water samples were collected from November 2011 to December 2014. The municipalities were selected based on leprosy epidemiology, as well as geological and climatic conditions. The municipalities had the following rates (new cases/10,000 inhabitants) of leprosy detected in 2012: 7.6 in Boa Viagem, 32.1 in Crato, 38 in Juazeiro do Norte, 0 in Mulungu, and 45.2 in Sobral (SESA 2016). The climate in each municipality is semi-arid and hot, except in Mulungu, which is slightly more humid.

**Fig. 1:**
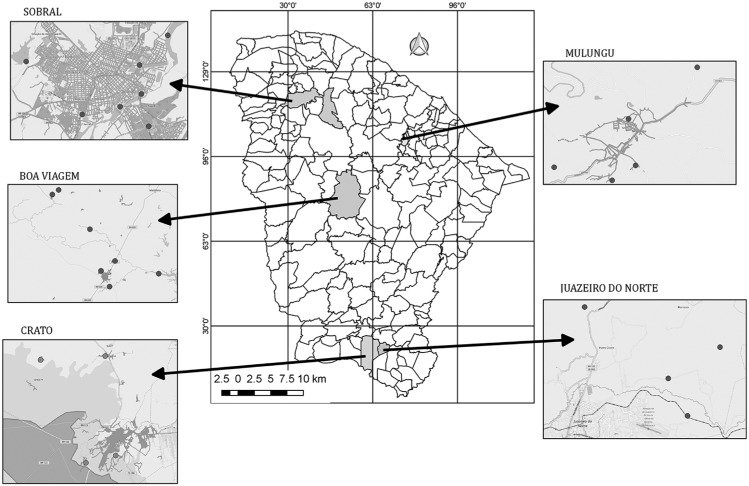
locations of water sources in the municipalities of Boa Viagem, Crato, Juazeiro do Norte, Mulungu, and Sobral, in the state of Ceará, Brazil. Grey dots (•) indicate the sites of water collection in each municipality.

The water samples were obtained from natural sources such as reservoirs, rivers, streams, springs, and wells. The collection points were selected by local health agents because they are used by the population for leisure and/ or domestic purposes (drinking, bathing, washing dishes, washing clothes, and tending animals). Samples were collected from five sites in Juazeiro do Norte, from eight sites in Sobral, and from four sites in Crato. In the municipalities of Boa Viagem and Mulungu, samples were collected from seven and six sites, respectively (Fig. 1).

*Water sample collection* - At each collection site, five samples were obtained from different depths identified by the letters “a” through “e” (surface, 25 cm, 50 cm, 75 cm, and 100 cm). In addition, the samples from each site were identified by the initial of the municipality [Boa Viagem (B); Crato (C); Juazeiro do Norte (J); Mulungu (M); and Sobral (S)], followed by the numerical order (B1 to B7; C1 to C4; J1 to J5; M1 to M6 and S1 to S8) and the replicates in alphabetical order (B1a, B1b, B1c, B1d, B1e, B2a … B7e). The waters were collected in a Van Dorn bottle, which allows the collection of water from the sub-surface to the bottom of a water source, trapping water at the selected depth. Immediately after water collection, physicochemical parameters (electrical conductivity, pH, and temperature) were measured. Water samples were then packed in sterile amber 1000-mL bottles and transported in styrofoam containers with ice within 24 h to the laboratory located in the capital of Ceará. During transport from the municipality to the capital city, one sample from the municipality of Boa Viagem (replica B7d) was damaged; the remaining 149 environmental samples from 30 sites were successfully delivered.

*Nucleic acid extraction* - The entire 1000-mL volume of each water sample was initially vacuum-filtered through a Mo Bio® filter with a sterile 0.22-μm membrane. Using two sets of sterile forceps, the filter membrane containing the sediment was rolled with the top side facing inward and placed into a tube containing glass beads from a Power Water RNA Isolation Kit (Mo Bio®). The total RNA was then extracted according to the manufacturer's instructions. Prior to recovering RNA, samples were incubated with DNase I stock enzyme (Mo Bio®) for 15 min at 25°C. Total RNA was stored at −80°C. A negative control for the RNA extraction was included with each set of water samples processed, using sterile distilled water in place of the environmental samples.

Standard care was taken to prevent degradation and contamination during all of the extraction and purification steps, which included the use of sterile disposables previously treated with diethyl pyrocarbonate (DEPC) and sterile buffers prepared with DEPC-treated water. We also used sterile filter tips in all procedures.

*RT-PCR of the M. leprae gyrA gene* - The PCR primers used to detect viable *M. leprae* were designed using the Primer3Plus program and targeted a 3750-bp ML0006 genomic region (gyrA) of *M. leprae*, GenBank accession No. Q57532. The 187-bp product, located between bases 7515 and 7702 of *gyrA,* was amplified with the primers gyrA-forward 5'-CCC GGA CCG TAG CCA CGC TAA GTC-3' and gyrA-reverse 5'-ATC GCT GCC GGT GGG TCA TTA-3'. The *gyrA* transcripts were selected because of their role as an essential bacterial enzyme. In addition, *M. leprae* codes for a homologue endoribonuclease (RNAseE) that plays a central role in mRNA degradation (Cole et al. 2001) and in the *gyrA* message (Unniraman et al. 2002).

Reverse transcriptase, followed by amplification, was performed using a OneStep RT-PCR Kit (Qiagen), with a 50 μL reaction consisting of 10 μL of 5× RT-PCR Buffer, 2.5 μL of total extracted RNA, 0.5 μL of each primer, 2 μL of RT-PCR Enzyme Mix, 2 μL of dNTP mix (10 mM), and a sufficient volume of RNase-free water. In each run, 20 pg of *M. leprae* DNA (extracted from a leprosy case by biopsy) was included as a positive control, and, as a negative control, we used 2.5 μL of RNase-free H_2_O in place of a nucleic acid template.

The reverse transcription reaction occurred at 50°C for 5 min, followed by denaturation at 95°C for 15 min. Amplification occurred with initial heating at 94°C for 5 min, followed by six cycles of denaturation at 94°C for 45 sec, annealing at 65°C for 30 sec, extension at 72°C for 90 sec, and then 35 denaturation cycles at 94°C for 45 min, annealing at 62°C for 45 sec, extension at 72°C for 45 sec, and a final extension at 72°C for 10 min. After amplification, the products were visualised by separation on a 1% (w/v) agarose gel stained with ethidium bromide solution. The fragments were visualised under ultraviolet light.

*Sequencing and alignment of M. leprae gyrA amplification products* - The PCR products were purified using a QIAquick PCR Purification Kit and then sequenced on an ABI 3130 Genetic Analyzer (Perkin-Elmer Applied Biosystems) using BigDye Terminator v3.1 Cycle Sequencing (Applied Biosystems) and BigDye XTerminator Purification (Applied Biosystems) kits. The sequences were converted to FASTA format using BioEdit Sequence Alignment v. 7.2.5, and then the basic local alignment search tool (BLAST) at NCBI (http://www.ncbi.nlm.nih.gov/) was applied. The taxonomic identities of the products were determined by comparison with the search results. The search result aligned our amplification products with the sequences of *M. leprae* BR4923 (GenBank accession No. FM211192), *M. leprae* TN chromosome (GenBank accession No. AL5839P7), *M. leprae* cosmid B1770 (GenBank accession No. Z70772), and *M. leprae* DNA gyrase subunit A gene (GenBank accession No. Z68206).

*Statistical analysis* - The data were entered into Microsoft® Excel 2013 and transferred to SPSS 16.0 statistical software (SPSS Inc., USA) to conduct descriptive and bivariate analyses. Fisher and Chi-square tests were used, with p values < 0.05 considered significant.

*Spatial analysis* - The home addresses of all new leprosy cases detected in Sobral in 2011 and the site of each of the water sources were georeferenced using Google Earth (https://www.google.com/earth/) to define latitude and longitude. After the exclusion of possible inconsistencies, such as sites located outside municipal boundaries and coordinates not determined because of incomplete addresses, the spatial database was visualised using QuantumGis Geographic Information System 18.1.0â, licensed by General Public License) (http://www.qgisbrasil.org). We used the georeferenced shape-file of municipalities in the state of Ceará (shapefile Arquive), available at the Brazilian Institute of Geography and Statistics [Instituto Brasileiro de Geografia e Estatística (IBGE 2016)], to prepare the maps. Based on the points formed by the geographic coordinates of the addresses of water source collection points in Sobral, Voronoi diagrams were created, with the polygons showing the proximity between cases and water sources. Geolocated residential addresses of leprosy cases in the other four municipalities were not available.

## RESULTS

Of the 30 water sources analysed, viable *M. leprae* was found in 23 (76.7%). Of the 30, there were two sources with five (6.7%) positive water samples, one source with four samples, eight (26.7%) sources with three and two positive samples, and four sources with only one (13.3%) positive sample. Regarding the municipalities, *M. leprae* was found in all sources in Juazeiro do Norte and Crato. In Sobral, six of the eight sources (75.0%) were positive. In Mulungu and Boa Viagem, *M. leprae* bacilli were found in four of six (66.7%) and four of seven (57.1%) sources, respectively (Table). No differences were found in positivity in relation to the type of source (reservoir, pond, spring, river, stream, or well). Similarly, no differences in positivity were found in relation to the type of water use (human, human/animal, irrigation, irrigation/ animal). Most of the water sources (27; 90%) were used for domestic purposes (drinking, bathing, cooking, washing clothes/dishes, recreational), with three sources used indirectly for humans, i.e., for irrigation and animal use.

**TABLE:** Distribution of *Mycobacterium leprae* mRNA positivity in the five municipalities of Ceará, Brazil, by type of source and purpose use of the water

Municipality	Place	Type of source	positive RNAm N (%)^a^	Purpose of use
Juazeiro do Norte (N = 25)	J1	dam	2 (40.0)	human
	J2	dam	2 (40.0)	human/animal
	J3	stream	2 (40.0)	human
	J4	dam	5 (100.0)	human
	J5	stream	4 (80.0)	human
		total	15 (60.0)	
Crato (N = 20)	C1	resort	2 (40.0)	human
	C2	dam	3 (60.0)	human
	C3	dam	3 (60.0)	human
	C4	dam	1 (20.0)	human/animal
		total	9 (45.0)	
Sobral (N = 40)	S1	dam	1 (20.0)	human
	S2	dam	0	human
	S3	lake	2 (40.0)	irrigation
	S4	river	3 (60.0)	human
	S5	river	3 (60.0)	human
	S6	stream	3 (60.0)	human
	S7	shallow river	0	human/animal
	S8	shallow river	2 (40.0)	human/animal
		total	14 (35.0)	
Boa Viagem (N = 34)	B1	dam	5 (100.0)	human
	B2	dam	0	human
	B3	dam	0	human
	B4	dam	3 (60.0)	human
	B5	dam	2 (40.0)	human/animal
	B6	dam	3 (60.0)	human
	B7	dam	0	human
		total	13 (38.2)	
Mulungu (N = 30)	M1	lake	0	animal/irrigation
	M2	well	1 (20.0)	human
	M3	well	1 (20.0)	human
	M4	well	2 (40.0)	human
	M5	stream	0	animal/irrigation
	M6	well	3 (60.0)	human
		total	7 (23.3)	

a: p = 0.0807, Chi-square test comparison of the mRNA positivity of the five municipalities.

*M. leprae* bacilli were detected in nine (30.0%) water samples collected at the surface, 11 (36.7%) samples each were found at 25-cm and 50-cm depth, 14 (46.7%) water samples were found at 75-cm depth and at 100-cm depth, and 15 (50.0%) samples at 100- cm depth were found.

The BLAST search results confirmed that the product sequences were those of *M. leprae gyrA.* The cDNA sequences from the water samples were aligned with the *M. leprae* cosmid Br4923 (FM211192), *M. leprae* Tamil Nadu strain (AL583917), *M. leprae* cosmid B1770 (Z70722), and *M. leprae* DNA gyrase subunit A gene (Z68206) sequences, with 99% and 100% similarities observed for all sequences.

Physicochemical parameters indicating quality were measured at the time of water sample collection (Supplementary data, Table). The mean electrical conductivity for all sources was 529.6 ± 344.5 μS/cm, with a mean of 574.2 ± 350.3 for water that was negative for the *M. leprae,* and 462.7 ± 314.6 for water that was positive. This difference was statistically significant (p = 0.044). Minerals were present, but no differences were found in electrical conductivity between the positive and negative samples from the five municipalities. No differences in electrical conductivity were found among water samples collected at different depths (surface to 1 m) from the same source. The mean temperature of all sources was 28.6 ± 2.5°C, with no difference between the positive (28.1 ± 2.2°C) and negative (28.8 ± 2.7°C, p = 0.156) samples. However, significant differences were observed between the temperatures of the positive (27.2 ± 0.5°C) and negative (28.3 ± 2.0°C; p < 0.025) samples from the municipality of Boa Viagem. According to the World Health Organization (WHO 2011), pH has no health impact on consumers, but it is considered as an important parameter indicating water quality. The pH of an ideal water source has been proposed to vary between 6.5 and 8.0. The studied water sources were alkaline, with a mean pH 7.8 ± 1.0. Only Sobral showed significant differences in pH in relation to *M. leprae* positivity, with a mean pH of 7.3 ± 0.4 in the positive samples and 8.3 ± 1.1 in the negative ones (p = 0.001).

*M. leprae* mRNA was found in water sources J4 and B1, in replicates collected at all depths, whereas samples S2, S7, B2, B3, B7, M1, and M5 all were negative. When comparing the mean values of the physicochemical parameters of sources J4 and B1 in and those of S2, S7, B2, B3, B7, M1, and M5, positive samples were observed to have a mean temperature of 28.3 ± 0.68°C, whereas the negative sources had a mean temperature of 29.4 ± 3.24°C (p = 0.015, t-test for independent samples). The same comparisons for electrical conductivity and pH between these positive and negative sources were conducted, but no significant differences were found (electrical conductivity, p = 0.055; pH, p = 0.120).

**Fig. 2:**
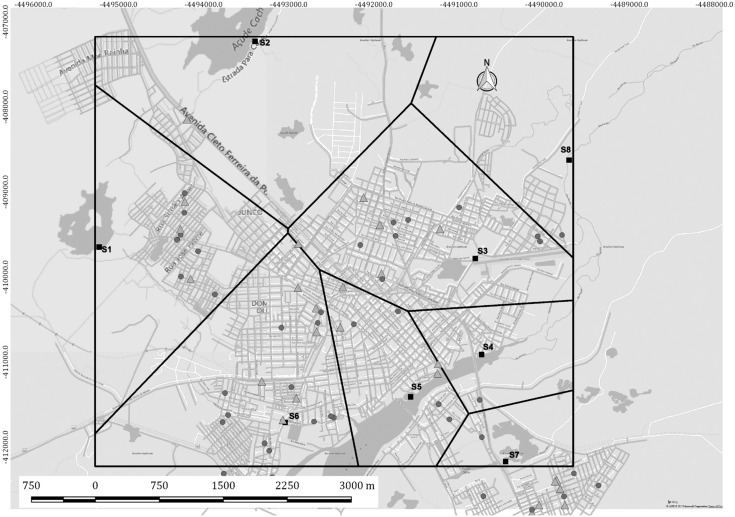
map of the city of Sobral, state of Ceará, Brazil, showing new leprosy cases detected in 2011 and sites where water was collected (S1 to S8). The grey squares (▪) indicate the water collection sites, black dots (•) show the residences of multibacillary cases, and the grey triangles (▵) indicate the residences of paucibacillary leprosy cases. Lines represent the Voronoi diagram.

Fig. 2 shows the map of the municipality of Sobral, with the location of the water sources analysed in this study and the residences of new MB and paucibacillary (PB) leprosy cases detected in year 2011. No viable *M. leprae* bacilli were found in samples S2 (reservoir) and S7 (river). These two sources were located far from the main river, and only one new case of leprosy was detected in their respective Voronoi polygons. In contrast, several new cases of MB and PB leprosy were detected close to sources S1, S3, S4, S5, S6, and S8. Points S1, S3, S4, S5, and S6 are located within the urban area of the municipality of Sobral. The main river runs towards the Atlantic Ocean, and therefore the bacilli present at point S6 could flow towards S5, then S4, and finally S8. At point S8 (positive for *M. leprae* mRNA), which is outside the city limits, only one new case of leprosy was detected in 2011.

## DISCUSSION

Leprosy is considered endemic in the northern and northeastern regions of Brazil. In 2015, in the state of Ceará, approximately 18.4% of municipalities were classified as hyperendemic, with more than 40 new cases per 100,000 inhabitants (SESA 2016). In many places, especially in remote areas of the northern and northeastern regions, access to health services is deficient, delaying the diagnosis of new cases and contributing to disease dissemination.

Several studies have suggested the role of the environment in disease transmission (Truman & Fine 2010, Mohanty et al. 2016). Our study is the first to report the presence of viable *M. leprae* in water sources in the American continent. The bacterium was identified in several natural water sources in five municipalities of the state of Ceará, Brazil. Viable bacilli were found in all five studied municipalities. Moreover, viable *M. leprae* was more frequently detected in municipalities with higher disease endemicity (Juazeiro do Norte, Crato, and Sobral) as compared to municipalities with lower endemicity (Boa Viagem and Mulungu). Other studies conducted in Asia have shown the presence of *M. leprae* DNA in rivers, wells, and lakes in a hyperendemic region in North Maluku, Indonesia (Matsuoka et al. 1999), and viable bacilli in sewers and wells of endemic regions in India (Mohanty et al. 2016). A recent study also conducted in the state of Ceará, Brazil, demonstrated the presence of genotype 4 *M. leprae* in environmental waters (Holanda et al. 2017). Other studies have shown the presence of viable bacilli in the peridomestic soil of leprosy cases in the Ghatampur and Purulia districts in India (Mohanty et al. 2016, Turankar et al. 2016). Future studies using genotyping techniques, including whole genome sequencing, are necessary to evaluate the similarity between the bacilli found in the environment and that found in patients.

Armadillos are known to be natural reservoirs of *M. leprae*, and contact with these animals is considered a risk factor for leprosy (Balamayooran et al. 2015, Kerr et al. 2015). In studies carried out in the southeastern United States, the same genotype of *M. leprae* was found in armadillos and human leprosy cases (Sharma et al. 2015). The study patients reported no direct contact with the animals, but they had direct contact with wild areas. In the state of Ceará, armadillos are commonly found in nature; hunting these animals is a leisure activity, and their meat is widely consumed in the countryside.

Studies carried out in Ceará, with animals captured at different sites, indicated that 21% of the animals carried *M. leprae* DNA (Frota et al. 2012), and another study in the state suggested that interaction with armadillos were a risk factor for transmission (Kerr et al. 2015). However, the role of the armadillo in this chain of disease transmission involving soil, water, humans, and other living beings is yet to be elucidated.

Northeastern Brazil has two climatic seasons, a dry season and a rainy season. The rains are concentrated in four months of the year, from February to May, and are influenced by the Intertropical Convergence Zone. However, the short rainy season has a highly variable intensity, influenced by the temperatures in the Atlantic Ocean. The countryside of Ceará is a semi-arid region, with rainfall intensity decreasing each year and temperatures reaching 40°C (Barroso et al. 2016). Therefore, the building of dams for water storage is common. In our study, 50% of the water sources analysed were reservoirs associated with dams. The few rivers found in the state of Ceará have shown reductions in volume annually or have become completely dry. Therefore, water sources are important and serve multiple purposes such as providing leisure activities, domestic use, and animal and agricultural use. Even in the wells in Mulungu, a mountainous region, people bathe and wash objects near the well. In our study, 90% of the water sources were heavily used by the local population for a variety of purposes. Patients with the MB form of the disease release bacilli into the environment through body secretions, and the bacillus can survive in the environment for a variable amount of time. Bacillus have been shown to remain viable up to 45 days and up to eight months within amoebas (Wheat et al. 2014). As demonstrated in Sobral, viable bacilli were found in water sources (S1 and S3 to S6) close to the domiciles of leprosy cases. Interestingly, sample S8, with only one case of the disease reported nearby, was also positive for *M. leprae.* This finding can be explained by the direction of the river flow in the municipality of Sobral. The river flows from west to east, going towards the Atlantic Ocean. The main river receives water from stream S6 and continues towards the Atlantic Ocean, with water sources found the following order downstream: S6, S5, S4, and S8. In the regions near sources S2 and S7 (negative for *M. leprae* mRNA), which are distant from the main river, only one case of leprosy was detected in each.

The finding of *M. leprae* in six water sources out of a total of eight in the municipality of Mulungu confirms what we suggested in the report of a study carried out with anti-PGL-1 in that population (Frota et al. 2010). Mulungu is a small isolated mountain town with a mild climate compared to that of other cities in the state of Ceará and with a leprosy prevalence of less than 1 case per 100,000 inhabitants. In our study conducted in 2010, seropositivity to anti-PGL-1 in Mulungu was 14%, similar to that found in the municipality of Sobral (endemic for leprosy), which was 15%. Mulungu is surrounded by five municipalities, which, in 2011, had case detection rates varying from zero to 53.4/100,000 inhabitants (DATASUS 2017). We suggest that leprosy detection is underestimated by the local government health agency and that this could have implications for the maintenance of disease in the region. In addition, we suggest that transmission of leprosy does not occur only through human contact, but that the environment also plays an important role in transmission of the disease. As suggested by another epidemiological study carried out in Ceará (Kerr-Pontes et al. 2006), bathing in natural water sources is a risk factor for leprosy.

*M. leprae* has been shown to survive in free-living pathogenic amoeba *in vitro* (Wheat et al. 2014). Furthermore, one study suggested that the ancestor of mycobacteria was an environmental organism living in an aquatic habitat (Ahmed et al. 2007). The association between viable *M. leprae* in water sources, particularly those used by the community, highlights the possible role of a protozoan or other organism in prolonging survival of the bacteria in the environment, including in soil and water.

We investigated the physicochemical properties of the collected water samples, and found that temperature and pH were only associated with the presence of live *M. leprae* only in Boa Viagem and Sobral. Positive water samples were also associated with significantly lower temperatures than those found in negative sources. However, it is difficult to infer the relevance of water temperature and the maintenance of the bacillus in these water sources.

Our results are consistent with the literature in associating water with leprosy cases. We emphasise the importance of demonstrating the presence of viable *M. leprae*. Future studies are necessary to demonstrate the linkage between *M. leprae* found in water and human infections through genotyping. We also suggest that early diagnosis of cases, along with the geolocation of residence, schools, and worksites, is important to monitor the emergence of new secondary cases.
