# Supplementary material for: Natural environmental water sources in endemic regions of northeastern Brazil are potential reservoirs of viable Mycobacterium leprae
Source: Mem Inst Oswaldo Cruz. 2017 Dec;112(12):805–11. doi: 10.1590/0074-02760170117 (PMC5719548; doi:10.1590/0074-02760170117)
Supplement: Supplementary file 1 [file 0074-0276-mioc-112-12-0805-Suppl01.pdf]

Mem Inst Oswaldo Cruz, Rio de Janeiro, Vol. *112*(12): 1-1, December 2017

TABLE

Physicochemical characteristics in the five municipalities of Ceará, Brazil, and the detection of *Mycobacterium leprae* mRNA

	Municipalities				
Physiochemical parameters (Mean ± SD)	Juazeiro do Norte (Mean ± SD)	Crato (Mean ± SD)	Sobral (Mean ± SD)	Boa Viagem (Mean ± SD)	Mulungu (Mean ± SD)
Electrical conductivity (µS/cm) (529.6 ± 344.5)					
Positive (462.7 ± 314.6)	347.2 ± 216.9	166.4 ± 231.8	581.2 ± 402.5	613.0 ± 77.3	536.9 ± 376.5
Negative (574.2 ± 350.3)	496.7 ± 254.8	318.4 ± 343.9	655.7 ± 421.5	572.5 ± 136.5	643.1 ± 396.6
Total	407 ± 262.4	250.0 ± 339.4	627.8 ± 433.7	588.7 ± 124.4	618.3 ± 418.2
p = 0.049	0.129	0.256	0.586	0.323	0.535
Temperature (ºC) (28.6 ± 2.6)					
Positive (28.1 ± 2.2)	27.5 ± 1.5	27.6 ± 2.4	31.1 ± 1.3	27.2 ± 0.5	26.5 ± 1.2
Negative (28.8 ± 2.7)	27.4 ± 1.1	28.7 ± 2.5	31.8 ± 2.4	28.3 ± 2.0	26.7 ± 1.1
Total	27.4 ± 1.5	28.2 ± 2.8	31.5 ± 2.2	27.8 ± 1.8	26.6 ± 1.2
p = 0.156	0.976	0.318	0.302	< 0.025	0.684
pH (7.8 ± 1.0)					
Positive (7.8 ± 0.9)	7.0 ± 0.7	7.5 ± 1.3	7.3 ± 0.4	8.7 ± 0.3	6.8 ± 0.6
Negative (7.9 ± 0.1)	8.2 ± 0.9	7.0 ± 1.1	8.3 ± 1.1	8.5 ± 0.4	7.0 ± 0.6
Total	8.1 ± 0.9	7.2 ± 1.3	7.9 ± 1.1	8.6 ± 0.4	7.0 ± 0.7
p = 0.585	0.416	0.435	0.001	0.114	0.346

SD: standard deviation.
